# The haplotype of UBE2L3 gene is associated with Hashimoto’s thyroiditis in a Chinese Han population

**DOI:** 10.1186/s12902-016-0098-6

**Published:** 2016-04-19

**Authors:** Yu Wang, Yuan-feng Zhu, Qiong Wang, Jing Xu, Ni Yan, Jian Xu, Liang-feng Shi, Shuang-tao He, Jin-an Zhang

**Affiliations:** Department of Endocrinology, The First Affiliated Hospital of Xi’an Jiaotong University Health Science Center, No. 277 West Yanta Road, Xi’an, 710061 China; Department of Endocrinology, Jinshan Hospital of Fudan University, No. 1508 Longhang Road, , Jinshan District Shanghai, 201508 China

**Keywords:** Ubiquitin conjugating enzyme E2L3 (UBE2L3), Single-nucleotide polymorphism (SNP), Autoimmune thyroid disease (AITDs), Graves’ disease (GD), Hashimoto’s thyroiditis (HT)

## Abstract

**Background:**

The ubiquitin conjugating enzyme E2L3 (UBE2L3) gene is associated with susceptibility to many autoimmune diseases. The aim of this study was to investigate the association between UBE2L3 gene and autoimmune thyroid diseases (AITDs) and their clinical phenotypes.

**Methods:**

We genotyped five single-nucleotide polymorphisms (SNPs) rs131654, rs5754217, rs2298428, rs140489 and rs5998672 of UBE2L3 gene in case groups including 1028 patients with AITDs [676 cases of Graves’ disease (GD) and 352 cases of Hashimoto’s thyroiditis (HT)] and control group including 897 healthy individuals.

The genotyping was performed with the method of polymerase chain reaction-ligase detection reaction (PCR-LDR).

**Results:**

The frequencies of allele and genotype of five SNPs in gene UBE2L3 showed no statistically significant difference between case groups and control group, respectively. Moreover, no significant differences in frequencies of allele and genotype of five SNPs of the gene were found between clinical subphenotypes of AITDs and control group. Such subphenotypes included GD, HT, and thyroid associated ophthalmopathy (TAO). The negative results were also found in the frequency of other haplotypes of the gene except the haplotype of TCGGC, which was significantly higher in HT group than in control group (*P* = 0.031, OR = 1.441).

**Conclusions:**

The present findings indicate that TCGGC haplotype is associated with an increased risk of HT and UBE2L3 gene is likely to be a susceptibility factor to HT in a Chinese Han population.

**Electronic supplementary material:**

The online version of this article (doi:10.1186/s12902-016-0098-6) contains supplementary material, which is available to authorized users.

## Background

Autoimmune thyroid diseases (AITDs) are a set of organ specific endocrine autoimmune diseases mediated by T lymphocyte. They mainly consist of Graves’ disease (GD), Hashimoto’s thyroiditis (HT), and thyroid associated ophthalmopathy (TAO). GD and HT are the most common phenotypes of AITDs. Now the pathogenesis of AITDs is thought to be immune dysfunction due to the interaction between genetic susceptibility and environmental factors. The recent studies have identified more than ten susceptibility loci for AITDs, which include cytotoxic T lymphocyte associated protein 4 (CTLA4) gene [1], thyroid stimulating hormone receptor (TSHR) gene [2], protein tyrosine phosphatase, non receptor type 22 (PTPN22) gene [3], interleukin (IL17) gene [4], thyroglobulin (TG) gene [5], human leukocyte antigen (HLA) gene [6], and FCRL3 gene [7]. The majority of susceptibility genes belong to immunity-related genes.

Ubiquintin proteasome pathway (UPP) is an important signal transduction system which involves many physiological processes. In this pathway, UBE2L3 is an ubiquitin conjugating enzyme (E2) participating in ubiquitination of substrate through sequential steps catalyzed together with activating (E1) and ligase (E3) enzymes, and exerts its degradation effects on targeting molecules [8]. These targeting molecules encompass p53 [9], c-Fos [10] and some immune or inflammatory reaction related molecules such as nuclear factor-κB (NF-κB) [11] and Toll-like receptors (TLR) [12].

Genetic variation in the UBE2L3 has been demonstrated in many autoimmune diseases such as systemic lupus erythematosus (SLE) [13–15], rheumatoid arthritis (RA) [15, 16] and Crohn’s disease (CD) [17, 18]. As we all know, many different autoimmune diseases often co-exist in one individual or aggregate in different members of one family. More importantly, accumulated evidence indicates that different autoimmune diseases may share common susceptible genes. Therefore, exploring the UBE2L3 gene mutation in the etiology of AITDs is a pivotal issue. In the present case–control study, we analyzed the SNPs in UBE2L3 region, rs131654, rs5754217, rs2298428, rs140489 and rs5998672 in AITDs in a Chinese Han population.

## Methods

### Subjects

We studied samples from Department of Endocrinology, the First Affiliated Hospital of Xi’an Jiaotong University Health Science Center, and Department of Endocrinology, Jinshan Hospital of Fudan University. The case groups including 1028 patients with AITDs cohort consisted of 676 in GD subgroup (208 males and 468 females, mean age of 36.918 years) and 352 in HT subgroup (44 males and 308 females, mean age of 34.774 years). The subjects in our study were not related to each other. Diagnostic basis for GD was typical high metabolic syndrome, different degrees of diffuse goiter, laboratory examination indicating hyperthyroidism and positive antibody against TSH receptor (TRAb). HT was confirmed by goiter, thyroid antibody against Tg (TgAb) or antibody against thyroid peroxidase (TPOAb) or even thyroid puncture pathological examination. The control group included 897 healthy controls (300 males and 597 females, mean age of 38.734 years), who were all screened for absence of thyroid goiter and personal or family history of thyroid diseases and any autoimmune diseases. The thyroid function was in the normal range. All subjects were ethnic Chinese Han and signed informed consent at each site. The study was approved by the Ethics Committee of Jinshan Hospital of Fudan University.

### Single-nucleotide polymorphism (SNP) genotyped of UBE2L3

We selected five SNPs rs131654, rs5754217, rs2298428, rs5998672 and rs140489 of UBE2L3 region because of their strong association with the other autoimmune diseases such as SLE, RA, Crohn’s disease, and Sjögren’s syndrome [13–18].

#### DNA extraction

The genomic DNA was extracted from 2 ml of peripheral blood from each subject using RelaxGene Blood DNA System (Tiangen biotech, Beijing, China) according to the manufacture’ s protocol.

#### Genotyping

UBE2L3 SNPs were genotyped using polymerase chain reaction-ligase detection reaction (PCR-LDR) and analyzed with polymerase chain reaction (PCR) machine (PRISM 3730, ABI). Five pairs of primers and the target gene LDR-probe sequences for SNPs of UBE2L3 region are shown as Additional file 1: Table S1.

### Clinical phenotype analysis

We analyzed the case groups’ clinical characteristics and correlation between frequencies of allele, genotype and haplotype and subphenotypes on the basis of [1] the onset age of disease (≤18 years versus ≥19 years); [2] presence or absence of AITDs family history (defined as the subjects’ first-degree relatives including parents, children and siblings or second-degree relatives such as grandparents, uncles and aunts who had AITDs occurrence); [3] presence or absence of ophthalmopathy (defined as a distinctive disorder characterized by inflammation and swelling of the extraocular muscles, eyelid retraction, periorbital edema, episcleral vascular injection, conjunctive swelling and proptosis); [4] euthyroid or hypothyroid HT (defined as serum TSH over 10 U/l) (Roche, China). The clinical characteristic data of the case groups are shown in Table 1.

**Table 1:** Clinical data of case groups and control group

	Control	AITDs	GD	HT
N	897	1028	676	352
Gender				
Male	300	252	208	44
Female	597	776	468	308
Onset disease of age				
≤ 18age		84	32	52
≥ 19age		501	223	278
Family history				
(+)		209	137	72
(−)		752	486	266
Ophthalmopathy				
(+)		126	120	6
(−)		764	486	278
Thyroid function				
Normal		244	116	128
Hyperthyroidism		445	445	0
Hypothyroidism		181	0	181

### Statistical analysis

Allele and genotype frequencies in case and control groups were implemented using SPSS17.0 (SPSS Inc., Chicago, IL, USA). Comparison of genotype and allele frequencies between case and control groups was made by Chi-square test. P value of < 0.05 was considered significant. Differences between groups were assessed by the odd ratio (OR) and 95 % confidence interval (95%CI). Haplotype frenquencies and linkage disequilibrium (LD) test were analyzed using Haploview 4.2 [19]. LD test was carried out using the pairwise LD analyze D’ and r^2^.

## Results

### Allele and genotype frequency distribution

There were no differences in allele or genotype frequency distribution in any SNP between patients (GD and HT) and control group (Additional file 1: Table S2). The same results were also found in subphenotypes such as GD patients with ophthalmopathy, family history and different thyroid function of HT patients (the data not shown).

### Haplotype frequencies analysis

**Fig. 1:**
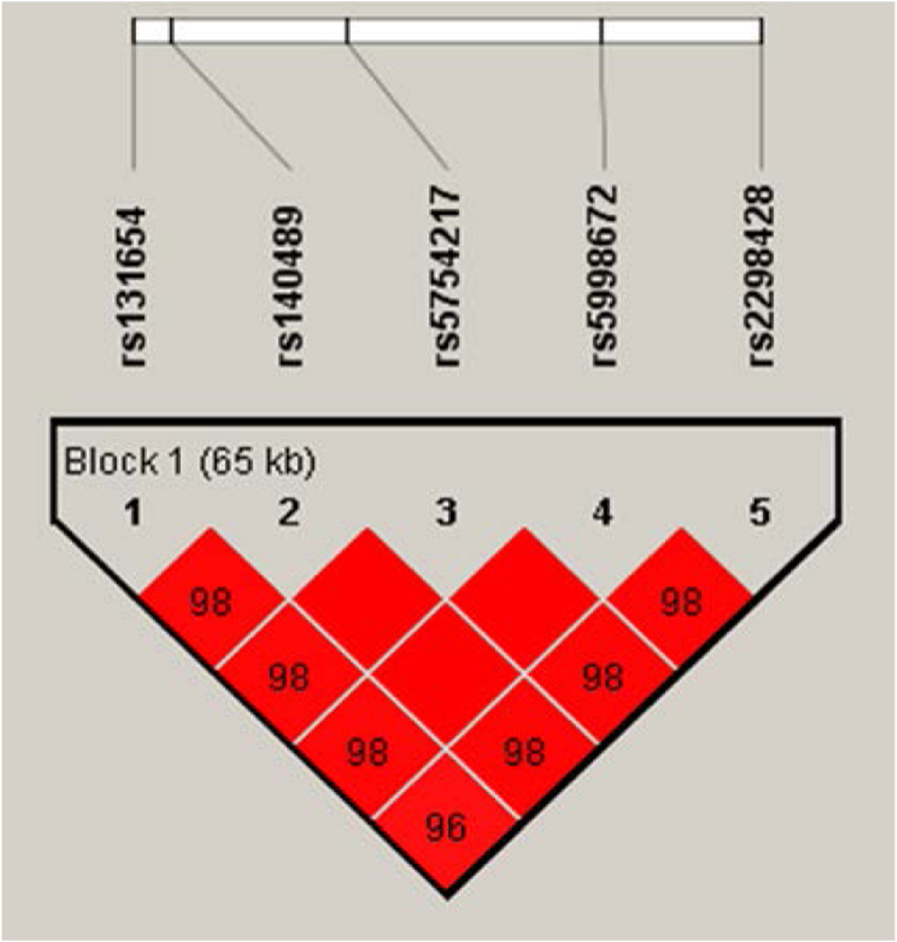
Five SNPs in the same LD block in UBE2L3 from the Hapmap CHB data

We found that five SNPs rs131654, rs5754217, rs2298428, rs5998672, and rs140489 of UBE2L3 region were in the same LD block (65 kilobases) using Hapmap CHB data (corresponding to samples of China ancestry from Beijing) and Haploview software. Strong linkage disequilibrium was shown for five SNPs (Fig. 1).

We obtained four haplotypes GCGGC, TTTAT, TCGGC and TTTAC in the UBE2L3 LD block using Haploview software, with frequencies all above 0.05. Unfortunately, compared with control group, the frequencies of haplotypes of GCGGC, TTTAT and TTTAC revealed no statistical difference with GD, HT or integrated AITD group respectively. However, the frequency of TCGGC haplotype in HT patients was significantly higher than that in healthy controls (*P* = 0.031, OR = 1.441), which suggested that the haplotype TCGGC increased the risk of HT (Table 2).

**Table 2:** Haplotype frequencies in case groups and healthy control group

Haplo type	Control (%)	AITD (%)	P	OR	95 % CI	GD (%)	P	OR	95 % CI	HT (%)	P	OR	95 % CI
GCGGC	825 (46.40)	942 (46.20)	0.938	0.995	0.876-1.130	610 (45.70)	0.702	0.973	0.844-1.121	331 (47.30)	0.674	1.038	0.872-1.238
TTTAT	671 (37.70)	738 (36.20)	0.343	0.938	0.822-1.071	494 (37.00)	0.681	0.970	0.838-1.122	245 (34.90)	0.202	0.888	0.740-1.066
TTTAC	150 (8.40)	185 (9.10)	0.475	1.086	0.876-1.361	125 (9.40)	0.363	1.122	0.875-1.439	60 (8.50)	0.912	1.017	0.744-1.393
TCGGC	105 (5.90)	149 (7.30)	0.081	1.258	0.972-1.629	91 (6.80)	0.299	1.166	0.873-1.558	58 (8.30)	0.031	1.441	1.033-2.012

## Discussion

Ubiquitin-mediated specific cellular protein degradation is involved in the regulation of many cellular processes, including cell cycle progression, differentiation, transcriptional adjustment, antigen presenting, receptor signal transduction, tumorigenesis and cell apoptosis [20, 21]. Increasing evidence suggests that UPP is closely related to the occurrence and progression of many autoimmune diseases. UPP’s influence on the immune system is embodied in both innate and adaptive immunities [22]. Under normal circumstances, the body’s innate immune system identifies and combines with intrusive pathogens by such receptors as toll-like receptor (TLR), RIG-1 receptor (RLR), and NOD receptors (NLR), and activates certain transcription factors such as NF-κB, interferon regulatory factor (IRF) family, subsequently inducing the release of various cytokines and inflammatory chemokines to get rid of pathogen from the body. It has been already demonstrated that innate immunity related TLR gene is one of genetic factors for AITDs [23, 24]. The influence of UPP on innate immunity is also controlled negatively by deubiquitylating enzymes (DUBs). As one of the DUBs, TNFAIP3 gene was also showed an association with the AITDs in our previous study [25]. So, studying the relationship between the polymorphisms of UBE2L3 and AITDs is pivotal and interesting. In our study, the frequency of haplotype TCGGC was higher in HT patients than in normal group, This suggests that the genotype which influences the pathogenesis of HT may exist in the vicinity of or in the haplotype itself. That is to say, UBE2L3 gene polymorphism is a predisposing factor for HT occurrence.

Adaptive immunity is generally mediated by T and B lymphocytes. UPP is involved in the regulation of T and B cell functions by adjusting T cell receptor (TCR) and B cell receptor (BCR) signaling transduction. There have been reports on the correlation of five SNPs in UBE2L3 with many T cell or B cell mediated autoimmune diseases, such as SLE [14, 15, 26], RA [15, 16], Crohn’s disease [16, 18], celiac disease [16], Sjögren’s syndrome (SS) and neonatal lupus erythematosus [27]. As a set of complex autoimmune diseases, not only T cell and B cell infiltrate in the lesioned thyroid tissues of AITDs, but also both innate and adaptive immunities may be implicated in its development. It did not come singly but in pairs in the present study, which revealed the relation between UBE2L3 gene variation and the pathogenesis of AITDs.

Although various subtypes of AITDs share common genetic factors and biomarkers, they indeed have their own specific manifestations and probably specific pathophysiolgic mechanisms. We speculated that different clinical types of AITDs may have different prominent alleles and genotypes. But we did not get the predicted result after stratifying the AITDs patients into GD, HT, and TAO ones, cases with positive family history or not, and HT in euthyroid or hypothyroid state.

Herein, our study provide an important clue that SNPs in UBE2L3 are involving in the pathogenesis of HT. Nevertheless, we have to admit this result may be influenced by sample size, different ethnic groups or even different research methods. Further research on UBE2L3 gene in AITDs of other races or even on UBE2L3 gene expression in AITDs is expected in the future.

## Conclusions

Thus, we may conclude that UBE2L3 gene variant may be implicated in HT in a Chinese Han population. Further investigations with other ethnic cohorts await to confirm the implication.

### Availability of data and materials

The dataset supporting the conclusions of this article is included within the article and its additional file.

## Additional file

Additional file 1:
**Table S1.** Annealing temperature, primer and probe sequences for the sequences analysis of UBE2L3 SNPs. Table S2. Allele and genotype frequencies in GD and HT patients. (DOC 103 kb)

## Abbreviations

AITDs: autoimmune thyroid diseases
CD: crohn’s disease
CTLA4: cytotoxic t lymphocyte associated protein 4
E1: ubiquitin activating enzyme
E3: ubiquitin ligase enzymes
GD: graves’ disease
HLA: human leukocyte antigen
HT: hashimoto’s thyroiditis
IL17: interleukin 17
NF-κB: nuclear factor-κB
PCR-LDR: polymerase chain reaction-ligase detection reaction
PTPN22: protein tyrosine phosphatase, non receptor type 22
RA: rheumatoid arthritis
SLE: systemic lupus erythematosus
SNPs: single-nucleotide polymorphisms
TAO: thyroid associated ophthalmopathy
TG: thyroglobulin
TgAb: thyroid antibody against Tg
TPOAb: antibody against thyroid peroxidase
TRAb: antibody against TSH receptor
TSHR: thyroid stimulating hormone receptor
UBE2L3: ubiquitin conjugating enzyme E2L3
UPP: ubiquintin proteasome pathway
