# Supplementary material for: The haplotype of UBE2L3 gene is associated with Hashimoto’s thyroiditis in a Chinese Han population
Source: BMC Endocr Disord. 2016 Apr 19;16:18. doi: 10.1186/s12902-016-0098-6 (PMC4837539; doi:10.1186/s12902-016-0098-6)
Supplement: Additional file 1: — Table S1. Annealing temperature, primer and probe sequences for the sequences analysis of UBE2L3 SNPs. Table S2. Allele and genotype frequencies in GD and HT patients. (DOC 103 kb) [file 12902_2016_98_MOESM1_ESM.doc]

| **Supplementary Table 1**. Annealing temperature, primer and probe sequences for the sequences analysis of UBE2L3 SNPs. | | | |
| --- | --- | --- | --- |
| SNP | Annealing temperature | Primer sequence | LDR probe |
| rs131654 | 56℃ | Forward-TCTTCCTTCTCCTGGACTAC | Modify: P-CATTGACCACATGCTATTCCTTTTTTTTTTTTTTTTTT-FAM |
|  |  | Reverse-TCCATGCTGACTGGAGGAAG | G: TTTTTTTTTTTTTTTTGGACTACCAGGCCCTTCTGCTCC |
|  |  |  | T: TTTTTTTTTTTTTTTTTTGGACTACCAGGCCCTTCTGCTCA |
| rs5754217 | 56℃ | Forward-CCAGGTCTTTACTGGATCTC | Modify: P-ACACCCCAAGTCACAGAATGTTTTTTTTTTTTTTTTTTTTTTTTTTTTTT-FAM |
|  |  | Reverse-ACCAAACCTCTAAGGATC | G: TTTTTTTTTTTTTTTTTTTTTTTTTTTTTAAGGATCCTGGATAGCAAAACC |
|  |  |  | T: TTTTTTTTTTTTTTTTTTTTTTTTTTTTTTTAAGGATCCTGGATAGCAAAACA |
| rs2298428 | 56℃ | Forward-TACCCCAGTGTGCCTCCCA | Modify: P-CTTTCTCTTGCTCTTGGGAGTTTTTTTTTTTTTTTTTTTTTTTTTTTTTTTTTT-FAM |
|  |  | Reverse-TCATGCAGCCGCTCCCAAGA | C: TTTTTTTTTTTTTTTTTTTTTTTTTTTTTTTTCGGCTGCGGTGAAGGCCCCGACG |
|  |  |  | T: TTTTTTTTTTTTTTTTTTTTTTTTTTTTTTTTTTCGGCTGCGGTGAAGGCCCCGACA |
| rs5998672 | 50℃ | Forward-GAGTCTAATGGGCACCTTTC | Modify: P-TTCCAACAAGTGAGGCTTGATTTTTTTTTTTTTTTTTTTTTTTTTTTTTTTTTTTTTTTTTTTTTTTTTTTTTTTTTTT-FAM |
|  |  | Reverse-GTGGAGTTCAAAGCCTTGCG | A: TTTTTTTTTTTTTTTTTTTTTTTTTTTTTTTTTTTTTTTTTTTTTTTTTTTTTTTTTTTTTTAATGGGCACCTTTCATGGTGATTAT |
|  |  |  | G: TTTTTTTTTTTTTTTTTTTTTTTTTTTTTTTTTTTTTTTTTTTTTTTTTTTTTTTTTTTTTTTTAATGGGCACCTTTCATGGTGATTAC |
| rs140489 | 50℃ | Forward-TACTAAGTGCCAGACCTCTC | Modify: P-TTAAAGAAAATAAAGCACTAGAGAGTTTTTTTTTTTTTTTTTTTTTTTTTTTTTTTTTTTTTTTTTTTTTTTTTTTTTT-FAM |
|  |  | Reverse-TGTTAATGCTAACGTATCCC | C: TTTTTTTTTTTTTTTTTTTTTTTTTTTTTTTTTTTTTTTTTTTTTTTTTTTTTTTTTTTTTTTTTTTTTTTTTTTTTTGTATCCCAGAGGATGGTTTCAAGGG |
|  |  |  | T: TTTTTTTTTTTTTTTTTTTTTTTTTTTTTTTTTTTTTTTTTTTTTTTTTTTTTTTTTTTTTTTTTTTTTTTTTTTTTTTTTT GTATCCCAGAGGATGGTTTCAAGGA |

**Supplementary Table 2**. Allele and genotype frequencies in GD and HT patients

| SNP/ Allele/ Genotype | | Control(%)  (%) | GD(%)  (%) | P | OR/ 95%CI  95%CI | HT(%)  (%) | P | OR/ 95%CI  95%CI |
| --- | --- | --- | --- | --- | --- | --- | --- | --- |
| rs131654 |  |  |  |  |  |  |  |  |
| Allele | T | 940(52.988) | 732(53.795) | 0.655 | 1.033 | 368(52.571) | 0.852 | 0.983 |
|  | G | 834(47.012) | 621(46.205) |  | 0.896-1.190 | 332(47.429) |  | 0.825-1.172 |
| Genotype | GG | 199(22.435) | 141(20.982) |  |  | 72(20.571) |  |  |
|  | GT | 436(49.155) | 339(50.446) | 0.778 |  | 188(53.714) | 0.351 |  |
|  | TT | 252(28.410) | 192(28.571) |  |  | 90(25.714) |  |  |
| rs5754217 |  |  |  |  |  |  |  |  |
| Allele | T | 845(47.419) | 635(47.247) | 0.924 | 0.993 | 310(44.286) | 0.159 | 0.881 |
|  | G | 937(52.581) | 709(52.753) |  | 0.862-1.144 | 390(55.714) |  | 0.739-1.050 |
| Genotype | GG | 251(28.171) | 178(26.488) |  |  | 103(29.429) |  |  |
|  | GT | 435(48.822) | 353(52.530) | 0.341 |  | 184(52.571) | 0.152 |  |
|  | TT | 205(23.008) | 141(20.982) |  |  | 63(18.000) |  |  |
| rs2298428 |  |  |  |  |  |  |  |  |
| Allele | T | 681(38.173) | 498(37.109) | 0.544 | 0.956 | 244(34.957) | 0.136 | 0.870 |
|  | C | 1103(61.827) | 844(62.891) |  | 0.826-1.106 | 454(65.043) |  | 0.725-1.045 |
| Genotype | CC | 344(38.565) | 258(38.405) |  |  | 146(41.834) |  |  |
|  | CT | 415(46.525) | 328(48.882) | 0.401 |  | 162(46.418) | 0.291 |  |
|  | TT | 133(14.910) | 85(12.668) |  |  | 41(11.748) |  |  |
| rs140489 |  |  |  |  |  |  |  |  |
| Allele | T | 844(47.630) | 628(47.147) | 0.790 | 0.981 | 309(44.269) | 0.132 | 0.873 |
|  | C | 928(52.370) | 704(52.853) |  | 0.851-1.131 | 389(55.731) |  | 0.732-1.042 |
| Genotype | CC | 248(27.991) | 176(26.426) |  |  | 103(29.516) |  |  |
|  | CT | 432(48.758) | 352(52.853) | 0.259 |  | 183(52.436) | 0.136 |  |
|  | TT | 206(23.251) | 138(20.721) |  |  | 63(18.052) |  |  |
| rs5998672 |  |  |  |  |  |  |  |  |
| Allele | G | 957(53.643) | 722(53.402) | 0.893 | 0.990 | 397(56.392) | 0.215 | 1.117 |
|  | A | 827(46.357) | 630(46.598) |  | 0.860-1.141 | 307(43.608) |  | 0.937-1.332 |
| Genotype | AA | 198(22.197) | 139(20.562) |  |  | 62(17.614) |  |  |
|  | AG | 431(48.318) | 352(52.071) | 0.339 |  | 183(51.989) | 0.192 |  |
|  | GG | 263(29.484) | 185(27.367) |  |  | 107(30.398) |  |  |
